# A Case of Alpha-Fetoprotein-Producing Adenocarcinoma of the Esophagogastric Junction in which Long-Term Survival Was Achieved by Means of Individualized Multidisciplinary Therapy

**DOI:** 10.1007/s12029-018-0078-3

**Published:** 2018-02-19

**Authors:** Kazuyuki Tanaka, Mikihiro Fujiya, Masami Ijiri, Keitaro Takahashi, Katsuyoshi Ando, Yoshiki Nomura, Nobuhiro Ueno, Shin Kashima, Takuma Goto, Junpei Sasajima, Takahiro Ito, Kentaro Moriichi, Yusuke Mizukami, Hiroki Tanabe, Toshikatsu Okumura

**Affiliations:** 1 Division of Gastroenterology and Hematology/Oncology, Department of Medicine, Asahikawa Medical University, 2-1 Midorigaoka-higashi, Asahikawa, Hokkaido 078-8510 Japan; 2 Inflammatory Bowel Disease Center, Sapporo Higashi Tokushukai Hospital, Sapporo, Japan; 3 Department of Gastroenterology, Asahikawa Kousei Hospital, Asahikawa, Japan

## Introduction

Carcinoma of the esophagogastric junction (EGJ) is a type of upper digestive cancer that occurs within 2 cm above or below the EGJ regardless of the histological type. Most cases of carcinoma of the EGJ are detected at an advanced stage and thus show a poor prognosis [1, 2]. The most frequent sites of lymph node metastasis in carcinoma of the EGJ differ from carcinomas in other portions of the gastric tract and esophageal cancers, and a thoracic approach is sometimes taken during surgery. For this reason, unusual procedures (including lymph node dissection) and reconstruction methods are frequently required [3–6]. To date, the therapeutic strategy has not been fully established for carcinoma of the EGJ.

Alpha-fetoprotein (AFP) is a useful tumor marker for primary hepatocellular carcinoma and yolk sac tumor [7]. Although AFP-producing cancer develops in the stomach, such tumors rarely developed at the EGJ. AFP-producing carcinoma is thought to have a high potential for metastasis, particularly liver metastasis [8–11] and thus show a poor prognosis [10, 12]. We herein report a case of AFP-producing carcinoma of the EGJ in which long-term survival was achieved through multidisciplinary therapy.

## Case Report

**Fig. 1:**
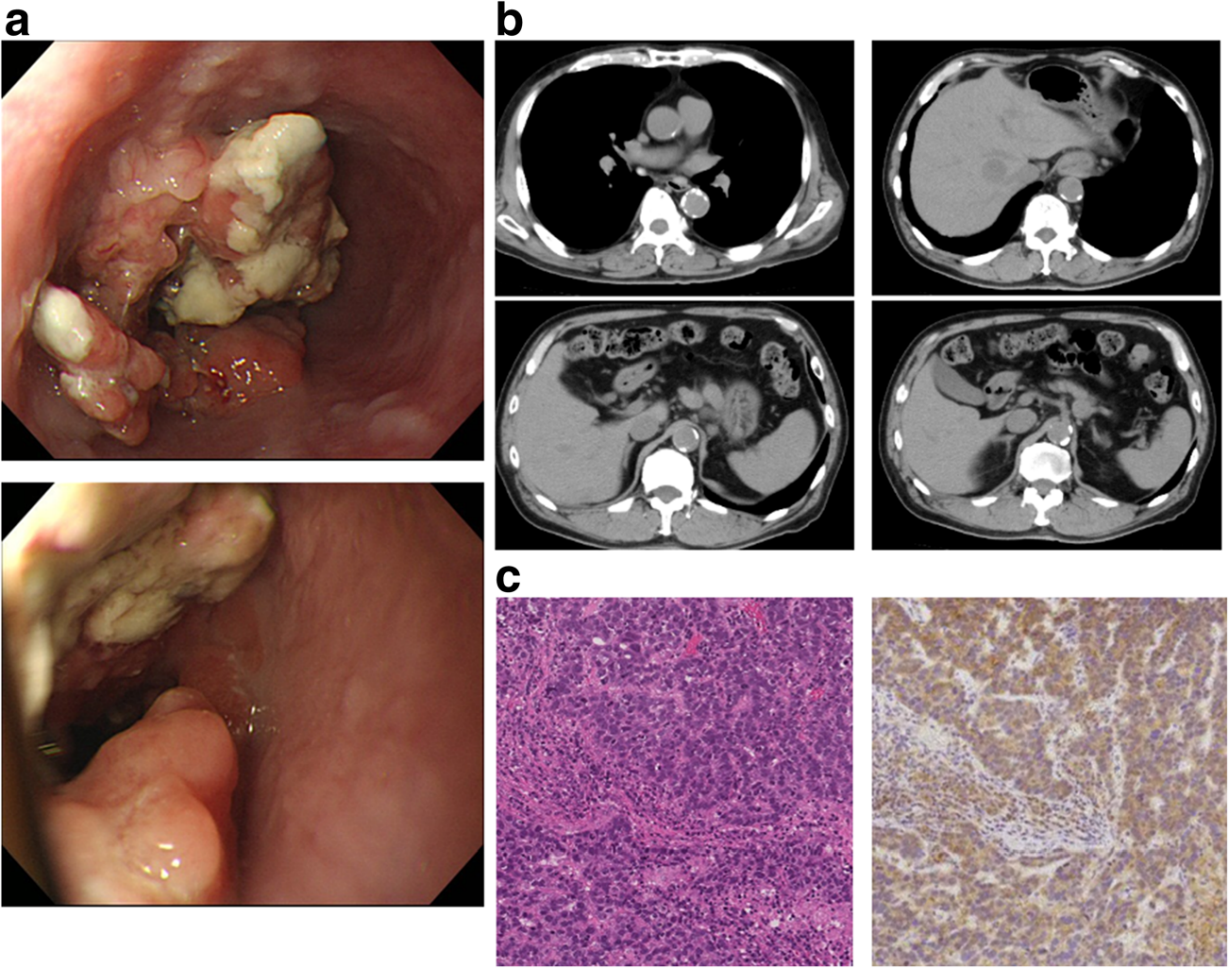
Endoscopic and computed tomography (CT) findings. **a** Esophagogastroduodenoscopy revealed a 3 cm-tumor with ulceration at the esophagogastric junction (EGJ). **b** Computed tomography showed multiple metastases in the lymph nodes and liver. **c** A histological examination revealed moderately to poorly differentiated adenocarcinoma with focal alpha-fetoprotein-positive cells in the EGJ

**Fig. 2:**
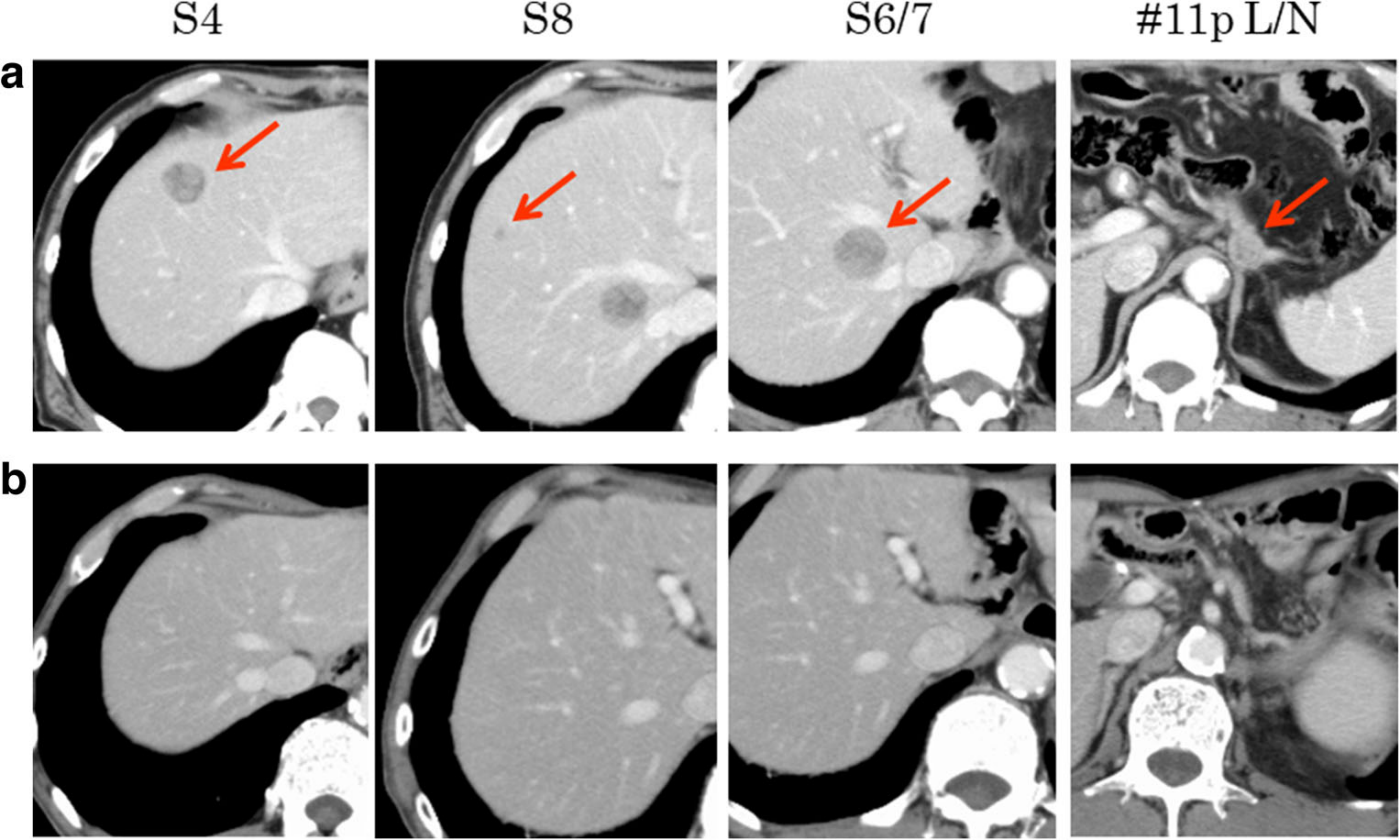
The changes in the CT findings during multidisciplinary therapy. **a** CT showed multiple metastases in the lymph nodes and liver. **b** After chemotherapy and percutaneous ethanol therapy (PEIT), all of the metastatic lesions disappeared

A 75-year-old man with hypertension, diabetes, and dyslipidemia underwent esophagogastroduodenoscopy to investigate the cause of a high CEA value and dysphagia in December 2010. An ulcerative circular tumor was detected at the EGJ (Fig. 1). Tubular adenocarcinoma cells were histologically detected in a tumor biopsy specimen. A laboratory analysis revealed mild anemia (hemoglobin 12.0 g/dL), renal dysfunction (creatinine 1.39 mg/dL, cystatin C 1.35 mg/L), and a high level of HbA1c (7.1%). An analysis of the patient’s serum revealed the following findings: squamous cell carcinoma (SCC), 3.4 ng/mL (normal range, < 1.5 ng/mL); carcinoembryonic antigen (CEA), 50.0 ng/mL (normal range, < 5 ng/mL); and carbohydrate 19-9 antigen (CA19-9), 1 U/mL (normal range, < 37 U/mL). An upper gastrointestinal series, which was performed for further confirmation, showed an irregular filling defect at the EGJ. The oral side of the lesion spread to the lower third of the esophagus; the stenosis was 3 cm in length. Computed tomography (CT) revealed wall thickening at the EGJ, multiple mediastinal and abdominal lymph node metastases, and multiple liver metastases in both lobes (Fig. 1). Magnetic resonance imaging (MRI) confirmed that the lesions were multiple liver metastases. Because the patient’s esophageal stenosis was symptomatic, total gastrectomy and lower esophagus resection with D2 lymph node dissection were performed. A histopathological examination showed the local production of AFP in moderately to poorly differentiated adenocarcinoma (Fig. 1). Due to the presence of the AFP-producing tumor, we checked the AFP level after surgery. At 568 ng/mL (normal range, < 5 ng/mL), the AFP level was elevated. First line chemotherapy with S-1 (100 mg/body on days 1 to 14) and docetaxel (50 mg/body on days 1 and 15) was administered in February 2011. While S-1 + cis-diamminedichloroplatinum (CDDP) therapy was recommended in the guidelines of the Japan Gastric Cancer Association, we hesitated to administer CDDP because of renal dysfunction due to diabetes. Therefore, S-1 + docetaxel (DOC) therapy was selected instead. Although grade 3 leukopenia and grade 2 anorexia and stomatitis were observed at the beginning of treatment, the therapy could be continued with a dose reduction. After 12 courses of chemotherapy, CT and contrast-enhanced ultrasonography revealed that only one lesion remained (S4) in the liver and that there was no lymph node metastasis. Because the liver metastatic lesion was close to vessels, chemotherapy and percutaneous ethanol therapy (PEIT) were selected in consideration of the patient’s safety and invasiveness. Maintenance chemotherapy with S-1 was then administered for 10 months. Thereafter, the patient’s serum AFP level decreased to the normal range. The patient has shown no recurrence in the 77 months since surgery (Fig. 2).

## Discussion

We reported a rare case of an AFP-producing carcinoma of the esophagogastric junction, in which long-term survival was achieved through multidisciplinary therapy that included surgery, chemotherapy, and PEIT. AFP-producing carcinoma of the gastrointestinal tract is considered to have a high potential for liver metastasis [8–11]. Carcinoma of the esophagogastric junction, particularly in advanced stages, is also known to have high malignant potential and shows a poor prognosis [1, 2]. It is notable that long-term survival was achieved in the present case despite the presence of two such unfavorable factors. The outcome suggests that our multidisciplinary approach is a practical option for the treatment tumors with such high malignant potential.

To date, eight cases exhibiting AFP-producing carcinomas of the EGJ have been reported in the literature (Table 1) [13–20]. Five cases were reported from Asia and three were reported from other regions. An association with Barrett’s esophagus was reported in half of the cases [13, 14, 16, 20], but in others no relationship was found [15, 17–19]. The histological subtypes were as follows: adenocarcinoma (*n* = 3) [16, 17, 19], hepatoid adenocarcinoma (*n* = 3) [14, 15, 18], adenocarcinoma/hepatoid adenocarcinoma (*n* = 1) [20], and mixed-type (*n* = 1) [13]. There were no cases of squamous cell carcinoma. Distant metastasis was frequently detected in cases with high serum levels of AFP at the diagnosis [14–16, 18–20]. The serum level of AFP appears to be useful as a marker of metastasis [17]. With regard to the prognosis, the present case achieved the highest survival term. In the present case, various treatments, including surgery, chemotherapy, and PEIT, were combined according to the patient’s condition because he had various complications, including renal failure and diabetes. PEIT is generally used for the treatment of hepatocellular carcinoma, while it is less commonly used for metastatic lesions from other organs, because most metastatic cases exhibit multiple lesions as well as frequent recurrences. However, the present case achieved the longest known survival period using PEIT, thus suggesting that personalized multidisciplinary therapy, including local treatments, such as PEIT, which are appropriate for the patient’s condition, is essential for the treatment of tumors with such a high malignant potential. The further accumulation of similar cases will be useful for establishing an appropriate therapeutic strategy for AFP-producing carcinomas that develop at the EGJ.

**Table 1:** The reported cases of AFP-producing adenocarcinoma of the EGJ with distant metastasis

Author	Year	Age	Sex	Pathological diagnosis	AFP (ng/mL)	Barrett	Distant metastasis	Therapy	Prognosis (duration)
Tanigawa H	2002	44	F	Hepatoid adenocarcinoma with choriocarcinoma and tubular adenocarcinoma	Normal	+	Liver	Surgery, chemotherapy	Dead (4 months)
Chiba N	2005	47	M	Hepatoid adenocarcinoma	326,400	+	Liver	Chemotherapy	Dead (14 months)
Fukuzawa J	2005	55	M	Hepatoid adenocarcinoma	47,800	–	Lung, bone	Surgery, chemotherapy	Dead (9 months)
Kripp M	2009	76	M	Adenocarcinoma (por)	5453	+	Liver, lymph node	Chemotherapy	Dead (18.5 months)
Chen YY	2013	45	M	Adenocarcinoma (tub2)	171.8	–	Lymph node	Surgery, chemotherapy	Dead (19 months)
Nagai Y	2014	62	M	Hepatoid adenocarcinoma (tub2 > por)	1473	–	Liver	Surgery, chemotherapy	Alive (25 months)
Haussler U	2016	61	M	Adenocarcinoma (por)	68,136	Not described	Liver	Chemotherapy	Alive (9 months)
Kashani A	2017	83	M	Adenocarcinoma (tub2) with hepatoid features	> 300,000	+	Liver, lung, lymph node	–	Dead (2 months)
Present case	2017	75	M	Adenocarcinoma (tub2)	568	–	Liver, lymph node	Surgery, chemotherapy, PElT	Alive (77 months)

## Abbreviations

*AFP*: alpha-fetoprotein
*EGJ*: esophagogastric junction
*CEA*: carcinoembryonic antigen
*SCC*: squamous cell carcinoma
*CA19–9*: carbohydrate 19-9 antigen
*CT*: computed tomography
*MRI*: magnetic resonance imaging
*PEIT*: percutaneous ethanol therapy
